# A statistical optimization for almost-complete methylene blue biosorption by *Gracilariabursa-pastoris*

**DOI:** 10.1016/j.heliyon.2024.e34972

**Published:** 2024-07-19

**Authors:** Ali Borham, Mohammed Haroun, Ibrahim A. Saleh, Naser Zomot, Mohammad K. Okla, Mofeed Askar, Mohamad Elmasry, Abdelmonem Elshahat, Lei Liu, Chen Zhao, Juanjuan Wang, Xiaoqing Qian

**Affiliations:** a Agricultural Products Safety and Environment, College of Agriculture, Yangzhou University, Yangzhou, 225127, China; b Key Laboratory of Cultivated Land Quality Monitoring and Evaluation, Ministry of Agriculture and Rural Affairs, Yangzhou University, Yangzhou, 225127, China; c Agricultural Botany Department, Faculty of Agriculture, Kafrelsheikh University, Kafr El-Sheikh, 33516, Egypt; d Faculty of Science, Zarqa University, Zarqa, 13110, Jordan; e Botany and Microbiology Department, College of Science, King Saud University, P.O. Box 2455, Riyadh, 11451, Saudi Arabia; f Economic Entomology Department, Faculty of Agriculture, Damietta University, Egypt; g Animal Production Research Institute (APRI), Agricultural Research Centre (ARC), Egypt; h Department of Horticulture, Faculty of Agriculture, Kafrelsheikh University, Kafr El-Sheikh, 33516, Egypt; i College of Environmental Science and Engineering, Yangzhou University, Yangzhou, 225127, China

**Keywords:** Dye removal, Plackett-burman design, Box-behnken design, Methylene blue, *Gracilaria bursa-Pastoris*, Biosorption, Seaweed

## Abstract

In this study, the dried biomass of four marine algae, namely *Porphyra* sp., *Gracilaria bursa-pastoris*, *Undaria pinnatifida* and *Laminaria* sp., were screened for their ability to remove methylene blue (MB) dye from aqueous solutions. Statistical approaches of the Plackett-Burman Design (PBD) and Box-Behnken Design (BBD) were applied to optimize different environmental conditions in order to achieve the maximum MB removal percentage by *Gracilaria bursa-pastoris*. The biosorbent was characterized before and after adsorption process using FTIR, XRD and SEM analysis. Additionally, isotherms, kinetics and thermodynamics studies were conducted to investigate the adsorption behavior of the adsorbent. The results showed that *Gracilaria bursa-pastoris* achieved the highest dye removal efficiency (98.5 %) compared to 96.5 %, 93.5 % and 93.9 % for *Undaria pinnatifida, Porphyra* sp. and *Laminaria* sp., respectively. PBD analysis revealed that the agitation speed, pH, and biomass dose were found to be the significant parameters affecting MB removal onto *Gracilaria* dried biomass. According to the BBD results, the maximum dye removal percentage (99.68 %) was obtained at agitation speed of 132 rpm, pH 7 and biomass dose of 7.5 g/L. FTIR, XRD and SEM analysis demonstrated the participation of several functional groups in the adsorption process and changes in the cell surface morphology of the adsorbent following the dye adsorption. The adsorption isotherms showed better fit to Freundlich model (R^2^ = 0.9891) than the Langmuir, Temkin, and Dubinin-Radushkevich models. The adsorption kinetics were best described by the pseudo-second-order model (R^2^ = 0.9999), suggesting the chemical interactions between dye ions and the algal biomass. The thermodynamic parameters indicated that the adsorption of MB onto *Gracilaria* dried biomass was spontaneous, feasible, endothermic and random. These results indicate that dried biomass of *Gracilaria bursa-pastoris* is an attractive, environmentally friendly, cheap and effective agent for MB dye removal from environmental discharges.

## 1 Introduction

Water pollution is a critical environmental issue that occurs when harmful substances contaminate streams, rivers, lakes, oceans, aquifers, and other bodies of water, leading to a deterioration of water quality and posing risks to human health and environment. The use of large quantities of commercial dyes is widespread across various industries such as textile industries, paper, cosmetic, pharmaceuticals, printing, food, leather, plastic etc. [1]. These industries produce substantial amounts of undesired colored wastewater that is heavily polluted with dyes and discharged as effluents [2]. The discharge of dye-containing effluents into water bodies increases biochemical and chemical oxygen demand, reduces sunlight penetration which can further impact the aquatic life. Additionally, Synthetic dyes are known to be recalcitrant, bio-accumulative, toxic, mutagenic, and carcinogenic [1,[3], [4], [5]].

Methylene blue (with the chemical formula C_16_H_18_N_3_SCl) is a heterocyclic aromatic basic dye. It's primarily utilized as a dyestuff in textile industry, additionally, it is employed in aquaculture for treating fungal infections. Despite its various beneficial uses, methylene blue dye can cause a range of negative effects on human beings such as increases heart rate, nausea, Heinz body formation, headache and gastritis. It also may lead to temporary or permanent eye burns and skin irritation [[6], [7], [8]]. Hence, the proper treatment of industrial textile effluents before their release into the environment is highly essential for the protection of both human health and the ecosystem. Various chemical and physical treatment methods have been used for dyes removal from aqueous solutions and wastewater effluents, such as oxidation, reduction, electrolysis, precipitation, ion-exchange, flocculation, filtration, adsorption [[9], [10], [11]]. However, these methods have disadvantages and restrictions in their application, as they are not consistently effective, are costly, and result in the production of substantial quantities of harmful solid wastes, which is not environmentally friendly [12,13].

Biological methods such as biodegradation, bioaccumulation and biosorption have been proved as efficient, low cost and eco-friendly approaches for dyes removal from textile wastewater [[14], [15], [16]]. Biosorption is an effective and low-cost method for removing dye and others pollutants from wastewater. Various natural materials such as, agricultural wastes and biomass of fungi and algae have been explored as biosorbents for their potential to remove dyes from effluents [17,18]. The process of biosorption using algal biomass is considered eco-friendly, cost-effective, and readily available, making it an attractive alternative to traditional wastewater treatment methods. Marine algae are highly effective for adsorbing a wide range of metals and dye pollutants in aqueous solutions, due to the presence of different functional groups such as carboxyl, hydroxyl, sulfate, and amino groups on their cell walls, which can bind to pollutants on the cell surface [19,20]. The diverse bioactive compounds and functional groups found in red and brown algae make them valuable and cost-effective in bioremediation [21,22]. The adsorption capacities of red and brown algae are higher than those of green algae, likely due to the main functional groups present in their cell walls [23]. The existence of alginates in the cell walls of brown algae and carrageenan in red algae is responsible for the binding of ions to algal biomass [24]. Different red seaweed species have been used in previous researche for dye removal. *Gracilaria edulis* was applied for adsorption of textile dye effluent [25] and Malachite green [26]. The dried biomass of *Gracilaria* sp. has been used for simultaneous removal of methylene blue and nickel from aqueous solutions [27]. Dried powder of *Pterocladia capillacea* was also applied for removing crystal violet dye from synthetic solutions [28].

Biosorption of dyes from aqueous solutions is indeed influenced by various parameters that affect the rate and efficiency of adsorption process. These factors include dye concentration, adsorbent dose, pH, temperature, static-agitation, and contact time [29,30]. These parameters play a crucial role in determining the efficiency of the biosorption process for removing textile dye effluent from aqueous solutions. Therefore, it's essential to optimize parameters to achieve the highest possible dye adsorption on the biosorbent. There are several methods available for optimization studies, ranging from basic such as one factorial at a time (OFAT) to more complex statistical designs such as Plackett-Burman design (PBD), Box-Behnken design (BBD), and Central Composite Design (CCD). The OFAT method involves varying one parameter at a time while keeping all other parameters constant. This method is relatively simple and easy to implement. However, it does not consider potential interactions between factors. Additionally, it requires a large number of experiments to optimize the process [31,32]. On the other hand, statistical designs, are multivariate approaches that allow for the simultaneous variation of multiple factors. These designs offer the advantage of providing a more comprehensive understanding of the experimental domain, allowing for the study of interactions between factors, and also require fewer experiments to optimize the process compared to the OFAT method [31]. Additionally, statistical designs provide higher-quality information within the experimental domain and can be used to evaluate the significance of factors in terms of their contribution to the response values [33]. The most commonly used statistical designs in scientific research for optimization studies are PBD, BBD and CCD, combine with Response surface methodology (RSM) [34,35].

To the best of our knowledge, there have been no reports conducted so far on the effectiveness of the red algae *Gracilaria bursa-pastoris* in dye removal. In the current study, the naturally powdered red algae *Gracilaria bursa-pastoris* was used as biosorbent for MB dye removal from aqueous solutions. The medium conditions were optimized to achieve the highest possible dye removal using the Plackett-Burman design and Box-Behnken design, combine with Response Surface Methodology. Also, Fourier Transform Infrared Spectroscopy (FTIR), X-ray diffraction (XRD) and Scanning Electron Microscope (SEM) were performed for characterization of the dried algal biomass before and after dye adsorption. Additionally, isotherms, kinetics and thermodynamics studies were applied to describe adsorption process.

## 2 Materials and methods

### 2.1 Preparation of algal biomass

The four seaweeds; *Porphyra* sp., *Gracilaria bursa-pastoris*, *Undaria pinnatifida* and *Laminaria* sp. were ordered from Fujian Yiyuan Co., Ltd (Fujian, China).

Algal biomass was washed initially with tap water many times to remove salts, sand and other contaminants that could be adhering to the surface. Then washed with distilled water 3 times. The clean biomass was then air dried at 70 °C for 48 h. The dried biomass was then crushed and grounded using an electric blender and then sieved manually to get a particle size less than 200 μm. The dried algal biomass was stored in desiccator until further use for biosorption experiments.

### 2.2 Preparation of dye solution

Methylene blue dye (MB) with molecular formula C_16_H_18_N_3_SCl was purchased from Sinopharm Chemical Reagent Co., Ltd (Shanghai, China).

The stock solution of MB (1000 mg/L) was prepared by the completely dissolving a weighed amount of dye in distilled water. Then, the required concentrations were prepared by dilution of the appropriate amount of stock solution in distilled water. The pH of the solutions was adjusted using 0.1 mol/L HCl or 0.1 mol/L NaOH.

### 2.3 Algae screening for MB removal

The dried algal biomass of *Porphyra* sp., *Gracilaria bursa-pastoris*, *Undaria pinnatifida* and *Laminaria* sp. was screened to select the most efficient in removing MB dye from aqueous solutions.

Biosorption experiments for the four algae were performed as batch mode by adding 250 mg of the powdered algal biomass in 50 mL Falcon tubes with working volume of 25 mL of MB solution (50 mg/L). All tubes were then shaken using an orbital shaker for 4 h at 150 rpm and ambient temperature. A 1 mL solution was withdrawn and centrifuged at 12000 rpm and 4 °C for 10 min. The MB removal percentage was assayed in the supernatant using spectrophotometer by measuring the absorbance at the wavelength of its maximum visible absorbance (*λ* Max 661 nm). All experiments were carried out in triplicates.

The following equation was used to calculate MB removal percentage (%):

(1) $$Y=\frac{\left(C_{i}-C_{f}\right)}{C_{i}}\times 100$$

where Y is the dye removal percentage (%); C_i_ is the initial dye concentration; and C_f_ is the final dye concentration.

The obtained data was subjected to statistical analysis using One Way ANOVA and means values were compared using Duncan Multiple Range Test (DMRT) at p < 0.05.

The dried algal biomass of *Gracilaria bursa-pastoris*, which showed the highest dye removal efficiency, was selected for further investigation.

### 2.4 Plackett-Burman design (PBD) for parameters screening

A Plackett-Burman design was employed to screen six independent parameters (initial dye concentration, pH, biomass dosage, temperature, agitation speed and time) and determine which significantly affect the removal of MB by the dried algal biomass of *Gracilaria bursa-pastoris*.

The six independent factors were studied at lower (−1), and higher (+1) levels (the range and levels are listed in Table 1).

**Table 1:** Actual and coded levels of independent factors tested using PBD.

Variables		Levels	
Name	Unit	−1	1
Dye concentration (X_1_)	mg/L	20	100
pH (X_2_)	–	3.5	9.5
biomass dosage (X_3_)	g/L	1	10
Temperature (X_4_)	°C	25	35
agitation speed (X_5_)	rpm	0	150
Contact time (X_6_)	h.	1	4

The following equation was used to create PBD based on the first-order polynomial model.

(2) $$Y=\beta_{0}+\sum \left(\beta_{i}X_{i}\right)$$

where Y represents the response variable (MB removal percentage), β_0_ is the intercept and β_i_ is the linear coefficient and X_i_ is the level of each independent variable.

The experimental matrix was generated by Design-Expert® (version 13.0.5.0.) using PBD with six factors included 12 experimental trials. All runs were carried out in triplicate, and the mean value was reported.

The factors that showed p-value <0.05 (confidence level >95 %) were considered as significant and were selected for further optimization using the Box-Behnken design of RSM.

### 2.5 Response surface methodology (RSM) using Box-Behnken design (BBD) for statistical optimization

After screening the six parameters using PBD to detect the shortlisted parameters that significantly affecting MB removal, the RSM using the BBD design was conducted to determine the optimal value of each factor for maximizing the dye removal, and to investigate their interactions.

The variables considered were agitation speed, pH and biomass dosage. These three independent variables (factors) were evaluated at three levels; −1, 0, and +1 (actual and coded levels are given in Table 2).

**Table 2:** Actual and coded levels of independent factors tested using Box– Behnken design.

Variables		Levels		
Name	Unit	−1	0	1
Agitation speed (X_1_)	rpm	0	75	150
pH (X_2_)	–	3.5	6.5	9.5
Biomass dosage (X_3_)	g/L	1	5.5	10

The dye removal percentage (response variable) and the interactions between the independent variables was investigated using the second-order polynomial quadratic model equation as follows:

(3) $$Y=\beta_{0}+\sum_{i}\beta_{i}X_{i}+\sum_{ii}{\beta_{ii}X}_{i}^{2}+\sum_{ij}\beta_{ij}X_{i}X_{j}$$

where Y denotes the predicted response (dye removal percentage), β_0_ is the constant coefficient (model intercept), β_i_, β_ii_, and β_ij_ represent the coefficients of the linear, quadratic, and interaction coefficients, respectively. X_i_, X_j_ are the independent variables.

The experimental matrix generated by the Design-Expert® software, included 15 runs, each run was carried out in triplicates, and the dye removal percentage (response values) were recorded as the mean values. Laboratory validation was conducted on the model to validate its prediction.

### 2.6 Characterization of the biosorbent

The dry biomass of *Gracilaria bursa-pastoris* before and after MB dye adsorption were analyzed using the FTIR spectroscopy (FTIR; Thermofisher Nicolet IS50, Waltham, MA, USA) to identify the surface functional groups involved in the dye adsorption process. The powdered samples were prepared by mixing with potassium bromide, and FTIR spectra were measured within the 4000–400 cm^−1^ range. X-ray diffraction spectrometry (XRD; Bruker D8 Advance) was carried out to investigate the crystalline and amorphous structures at U = 40 kV and 1 = 30 mA. The surface morphology of the powdered *Gracilaria bursa-pastoris* before and after MB adsorption was studied using scanning electron microscopy (SEM; Thermofisher Quanta 250FEG).

### 2.7 Adsorption isotherms investigations

In the current study, the Freundlich, Langmuir, Temkin, and Dubinin-Radushkevich models were employed to describe the interaction between MB dye and the dried biomass of *Gracilaria*.

Adsorption isotherms study was performed at initial dye concentrations ranging from 20 to 100 mg/L, biomass dosage 10 g/L, pH 7.5, agitation speed 150 rpm, and contact time 4 h. The solution was centrifuged at 12000 rpm for 10 min and the supernatant was then measured spectrophotometrically for the final dye concentration. The following equation was used to calculate the amount of adsorbed dye (mg/g):

(4) $$q_{e}=\frac{\left(C_{i}-C_{f}\right)\times V}{m}$$

where q_e_ is the equilibrium adsorption capacity (mg/g); C_i_ and C_f_ are the initial and final dye concentrations (mg/L), respectively; V is volume of dye solution (L); and m is the mass of the adsorbent (g).

The Langmuir isotherm model assumes that adsorption occurs as a monolayer onto the adsorbent's surface. It further assumes that the adsorbent's surface is uniform with binding sites that have the same activation energy, in other words, all adsorption sites possess a similar affinity for the adsorbate [36]. The Langmuir isotherm model is described by Equation (5)

(5) $$\frac{1}{q_{e}}=\left(\frac{1}{K_{L}Q_{m}}\right)\;\frac{1}{C_{e}}+\left(\frac{1}{Q_{m}}\right)$$

where q_e_ is the amount of dye adsorbed on adsorbent (mg/g). C_e_ is the equilibrium concentration of dye (mg/L), K_L_ is the Langmuir constant (L/mg), and Q_m_ = maximum adsorption capacity (mg/g).

The separation factor R_L_ was calculated using Eq. (6):

(6) $$R_{L}=\frac{1}{1+C_{i\;}K_{L}}$$

where C_i_ is the initial dye concentration (mg/L).

Freundlich isotherm model suggests that the adsorption process occurs on heterogeneous surfaces with different sites having different adsorption energies, and the adsorbate is adsorbed on multilayers on the adsorbent's surface [37]. The Freundlich isotherm model is described by Equation (7)

(7) $${\text{logq}}_{e}=\frac{1}{n}{\text{logc}}_{e}+{\text{logK}}_{F}$$

where the intercept log K_F_ is the adsorption capacity (mg/g) and the slope 1/n is the adsorption intensity.

The Temkin isotherm model suggests that the adsorption energy of the molecules decreases linearly, not logarithmically, with surface coverage due to interactions between the adsorbate and the surface [38]. It can be expressed in Equation (8)

(8) $$q_{e}=\left(\frac{\text{RT}}{B}\right)\mathrm{lnA}+\left(\frac{\text{RT}}{B}\right)\;{\text{lnC}}_{e}$$

where A is the Temkin isotherm equilibrium binding constant (L/g), B is Temkin isotherm constant related to the heat of adsorption, R is the universal gas constant (8.314 J/mol K), and T is the absolute temperature in Kelvin (K).

Dubinin-Radushkevich isotherm is an empirical adsorption model that assumes a physical adsorption mechanism of gases and vapors with Gaussian energy distribution on microporous sorbents with heterogeneous surfaces [39]. It's described by Equation (9)

(9) $${\text{lnq}}_{e}={\text{lnq}}_{s}-K_{\text{DR}}\varepsilon^{2},\mathrm{where}\;Ɛ=\mathrm{RTln}\left[1+\frac{1}{C_{e}}\right]$$

where q_s_ is the theoretical adsorption capacity (mg/g), K_DR_ is the Dubinin-Radushkevich isotherm constant (mol^2^ kJ^−2^), and Ɛ is the Polanyi potential. The following Equation (10) was uesd to calculate the free energy of adsorption (E) for determining the adsorption nature (whether it is physisorption or chemisorption)

(10) $$E=\frac{1}{\sqrt{2K_{\text{DR}}}}$$

### 2.8 Adsorption kinetics

Adsorption kinetic models are used to monitor the rate at which the process occurs and provide insight into the mechanism of adsorption processes occurring onto the biosorbent.

Adsorption kinetic study was carried out at various time intervals (from 0 to 160 min) and different initial dye concentrations (20, 60 and 100 mg/L). After dye adsorption, equilibrium adsorption capacity (qe) was assessed and the data were fitted with pseudo-first- and second-order kinetic models.

The most commonly used kinetic models; Lagergren's pseudo-first-order model (Equation (11)) [40] and pseudo-second-order model (Equation (12)) [41], were used in this study to describe the adsorption mechanism of MB dye onto *Gracilaria*.

(11) $$\log\;\left(q_{e}-q_{t}\right)=-\;\frac{K_{1}t}{2.303}+{\text{logq}}_{e}$$

where q_e_ (mg/g) and q_t_ (mg/g) are adsorbed dye amount at equilibrium and contact time t (min), respectively; K_1_ is the rate constant of the first- order adsorption (min^−1^) which can be determined experimentally from the slope by plotting log (q_e_−q_t_) versus t.

(12) $$\frac{t}{q_{t}}=\frac{t}{q_{e}}+\frac{1}{K_{2}q_{e}^{2}}$$

where K_2_ is the is the rate constant of the second - order adsorption (g/mg· min). q_e_ and K_2_ were calculated by plotting t/qt versus t from the slope and intercept, respectively.

### 2.9 Adsorption thermodynamics

Thermodynamics study was carried out at temperature from 298 to 338 K with initial dye concentration of 100 mg/L, and contact time 4 h.

The following equations were used to calculate the thermodynamics parameters: Gibbs free energy change (ΔG°, K J/mol), enthalpy change (ΔH°, J/mol), and entropy change (ΔS°, J/mol K).

(13) $$K_{d}=\frac{q_{e}}{C_{e}}$$

(14) $$\Delta G{}^{\circ}=-\mathrm{RT}\;\ln\;K_{d}$$

(15) $$\ln\;K_{d}=\frac{\Delta S{}^{\circ}}{R}-\frac{\Delta H{}^{\circ}}{\text{RT}}$$

were K_d_ is equilibrium constant, R is the universal gas constant (8.314 J/mol K), T is the absolute temperature in Kelvin (K). The ΔH° and ΔS° values were calculated from the slope and intercept of plot ln K_d_ versus 1/T.

## 3 Results and discussion

### 3.1 Algae screening for MB removal

Dried biomass of four marine algae; *Porphyra* sp., *Gracilaria bursa-pastoris*, *Undaria pinnatifida* and *Laminaria* sp. were screened for their ability to remove MB from aqueous solutions in order to select the most efficient one in dye removal.

**Fig. 1:**
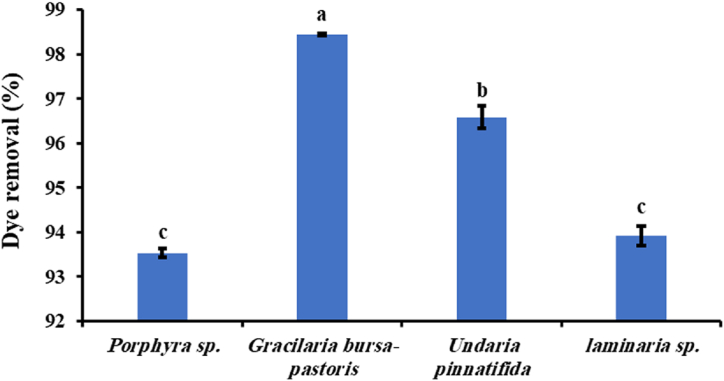
Marine algae screening for MB removal (dye concentration: 50 mg/L, biomass dosage: 10 g/L, contact time 4 h, agitation speed: 150 rpm). Bars with same letter are not statistically different according to the Duncan test at P < 0.05.

The data presented in Fig. 1 indicates the effectiveness of these four algae in removing MB dye. The dried biomass of *Gracilaria bursa-pastoris* showed the highest decolorization efficiency with a 98.5 % dye removal percentage, followed by *Undaria pinnatifida*, which revealed a dye removal percentage of 96.5 %. Whereas both *Porphyra* sp. and *Laminaria* sp. are not statistically different as they exhibited dye removal percentages of 93.5 % and 93.9 %, respectively. (The p-value is 0.0000).

### 3.2 Plackett-Burman design (PBD) for parameters screening

PBD serves as an efficient screening method to determine the significant parameters influencing the dye removal from numerous candidate parameters. This method is based on a first-order polynomial equation without interactions between the independent factors [42,43].

In this study, the six independent factors which are initial dye concentration, pH, biomass dosage, temperature, agitation speed and time were investigated by PBD to determine the significant factors affecting MB dye removal onto the dried algal biomass of *Gracilaria bursa-pastoris*.

PBD matrix with a total of 12 runs for screening the significant parameters influencing the MB dye removal onto *Gracilaria bursa-pastoris* were presented in Table 3.

**Table 3:** PBD matrix for selecting the significant parameters that affect MB dye removal.

Run	Variables						Dye Removal percentage (%)			
	Dye Concentration (X_1_)	pH (X_2_)	Biomass dosage (X_3_)	Temperature (X_4_)	Agitation speed (X_5_)	Time (X_6_)	Experimental^a^	Predicted	Residual	
**1**	100	3.5	10	35	150	1	93.66 ± 0.00	95.08	−1.41	
**2**	20	3.5	1	35	0	4	78.86 ± 3.99	80.05	−1.19	
**3**	20	9.5	10	35	0	1	94.71 ± 0.00	92.40	2.31	
**4**	20	3.5	1	25	0	1	75.22 ± 0.74	76.99	−1.77	
**5**	100	9.5	10	25	0	1	89.47 ± 0.08	91.06	−1.59	
**6**	100	9.5	1	35	150	4	97.40 ± 0.66	98.45	−1.05	
**7**	100	3.5	10	35	0	4	86.95 ± 0.37	86.26	0.69	
**8**	20	3.5	10	25	150	4	93.46 ± 0.88	91.60	1.86	
**9**	20	9.5	1	35	150	1	97.37 ± 0.63	96.72	0.65	
**10**	100	9.5	1	25	0	4	88.11 ± 1.60	86.57	1.54	
**11**	20	9.5	10	25	150	4	97.59 ± 0.22	99.45	−1.86	
**12**	100	3.5	1	25	150	1	89.34 ± 0.12	87.52	1.82	

a Experimental results are mean of triplicate ± standard deviation.

Multiple regression analysis was applied to the experimental results of the PBD and fitted to a first-order polynomial equation (Equation (16)) which describes the relationship between MB dye removal and the independent factors.

(16) Y = + 90.18 + 0.6443 X_1_ + 3.93 X_2_ + 2.46 X_3_ + 1.31 X_4_ + 4.62 X_5_ + 0.2172 X_6_

Where Y is the response (MB dye removal percentage) and X_1_, X_2_, X_3_, X_4_, X_5_ and X_6_ are the coded levels of the dye concentration, pH, biomass dose, temperature, agitation speed and time, respectively.

The analysis of variance (ANOVA) of the PBD presented in Table 4 showed that the model is significant with a p-value of 0.0043. p-value less than 0.05 indicates that the model is significant [27]. Also, the F-value of 15.53 for the model indicates its significance, which means there is only a 0.43 % chance that an F-value this large could occur due to noise [44]. The results also indicated that agitation speed followed by pH of the solution then biomass dose, had significant positive effects on MB dye removal with p-values of 0.0012, 0.0024 and 0.0166, respectively.

**Table 4:** Analysis of variance (ANOVA) for the PBD experimental results for MB dye removal.

Source	Sums of squares	df	Mean square	F-value	p-value	Remarks
**Model**	540.9400	6	90.1600	15.5300	0.0043	Significant
X_1_	4.9800	1	4.9800	0.8582	0.3968	Not significant
X_2_	185.2800	1	185.2800	31.9200	0.0024	Significant
X_3_	72.7500	1	72.7500	12.5300	0.0166	Significant
X_4_	20.7300	1	20.7300	3.5700	0.1174	Not significant
X_5_	256.6400	1	256.6400	44.2100	0.0012	Significant
X_6_	0.5663	1	0.5663	0.0975	0.7674	Not significant
**Residual**	29.0300	5	5.8100			
**Cor Total**	569.9700	11				
**Std. Dev.**	2.4100	**R**^**2**^**=**		0.9491		
**Mean**	90.1800	**Adj. R**^**2**^**=**		0.8880		
**C.V. %**	2.6700	**Pred. R**^**2**^**=**		0.7067		
		**Adeq. Precision**		12.2086		

**Std. Dev.:** standard deviation; **C.V.:** Coefficient of variation; **Adeq. Precision:** adequate precision.

**Fig. 2:**
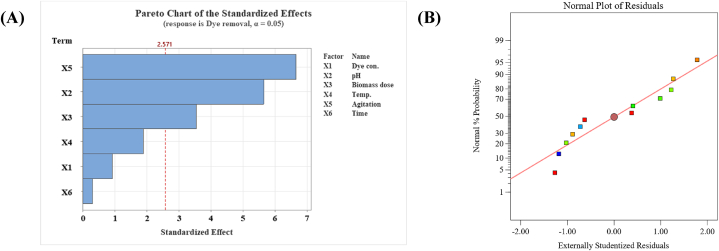
Pareto chart of the PBD results **(A)**, and Normal probability plot of the residuals generated by the first-order polynomial equation **(B)** for MB dye removal by *Gracilaria bursa-pastoris*.

These results were verified by the Pareto chart illustrated in Fig. 2A, which was employed to evaluate the standardized effect of individual factors on the dye removal.

The significance effect of each tested factor was identified at the 0.05 level (confidence level >95 %) using the red reference line on the Pareto chart [45]. As can be observed, agitation speed (X5) showed the most significant factor followed by solution pH (X2) then biomass dosage (X3), whereas both of dye concentration (X1), temperature (X4), and time (X6) had no significant effect on the dye removal.

The R^2^ value (coefficient of determination) of this model was 0.9491 meaning that 94.91 % variability in MB dye removal could be calculated by the model, leaving only 4.81 % unexplained.

The Predicted R^2^ of 0.7067 is in reasonable agreement with the Adjusted R^2^ of 0.8880; i.e. the difference is less than 0.2, demonstrating the accuracy of the model in dye removal predictions.

The normal probability plot of the residuals was presented in Fig. 2B. It's obvious that the residuals points are either on or close to the 45-degree straight line which indicate the residuals are normally distributed and there was no potential outlier detected in the data, demonstrating the PBD model was well fitted with the experimental data [46].

### 3.3 Response surface methodology (RSM) using Box-Behnken design (BBD) for statistical optimization

Box– Behnken design with response surface methodology involve a set of mathematical and statistical tools for optimization process [47,48].

Based on PBD results, the significant factors; agitation speed, pH and biomass dose, were selected for further statistical optimization using Box Behnken design (BBD) of response surface methodology (RSM).

The experimental design matrix of BBD containing these three factors with their three levels (−, 0, +) and the response values (dye removal percentage) were presented in Table 5. The other three factors; dye concentration, temperature and time which were insignificant on dye removal, were maintained at their high levels of PBD (which exerted a positive effect on dye removal).

**Table 5:** Box–Behnken design matrix and corresponding results for optimization of MB dye removal onto *Gracilaria bursa-pastoris* (dye concentration: 100 mg/L, temperature: 35 °C, contact time: 4 h, agitation speed: 150 rpm).

Run	Variables			Response Dye removal percentage (%)		Residuals
	Agitation speed (X_1_)	pH (X_2_)	Biomass dosage (X_3_)	Experimental^a^	Predicted	
**1**	0	3.5	5.5	89.22 ± 0.04	89.14	0.08
**2**	0	9.5	5.5	92.50 ± 0.18	92.54	−0.04
**3**	75	6.5	5.5	98.88 ± 0.04	98.85	0.03
**4**	75	6.5	5.5	98.88 ± 0.23	98.85	0.03
**5**	75	3.5	10	93.15 ± 0.66	93.25	−0.10
**6**	75	9.5	10	98.37 ± 0.08	98.35	0.02
**7**	0	6.5	1	92.58 ± 1.77	92.64	−0.06
**8**	75	9.5	1	98.02 ± 0.27	97.92	0.10
**9**	0	6.5	10	88.26 ± 0.04	88.25	0.02
**10**	150	9.5	5.5	99.32 ± 0.07	99.39	−0.08
**11**	75	6.5	5.5	98.80 ± 0.15	98.85	−0.06
**12**	150	6.5	10	98.28 ± 0.08	98.22	0.06
**13**	75	3.5	1	96.91 ± 0.43	96.93	−0.02
**14**	150	3.5	5.5	96.74 ± 0.35	96.70	0.04
**15**	150	6.5	1	97.07 ± 0.29	97.09	−0.02

a Experimental results are mean of triplicate ± standard deviation.

The results indicated notable differences in the dye removal percentages, ranging from 88.26 % to 99.32 %. The maximum value was achieved in run no. 10 with dye removal percentage of 99.32 % at the agitation speed of 150 rpm, pH 9.5 and 5.5 mg/L biomass dose. Whereas the minimum dye removal percentage of 88.26 % was recorded in run no. 9 where agitation speed was 0 rpm, pH was 6.5 and biomass dose was 10 mg/L.

The analysis of variance (ANOVA) for the quadratic model of BBD data was illustrated in Table 6. Based on F-value and p-value, the model is highly significant for the dye removal prediction. The Model F-value of 2185.05 implies the model is statistically significant. There is a mere 0.01 % probability that such a large F-value could occur by chance. The p-value of this model is less than 0.0001, which indicates its high significance. The Lack of Fit F-value of 5.82 suggests the Lack of Fit is not significant compared to the pure error. There is a 15.02 % chance that a Lack of Fit F-value this large could occur due to noise. Non-significant lack of fit indicates that the model is well-fitted [13,46].

**Table 6:** Analysis of variance (ANOVA) for the Box–Behnken Design experimental results.

Source	Sums of squares	df	Mean square	F-value	p-value	Remarks
**Model**	190.1000	9	21.1200	2185.0500	<0.0001	Significant
X_1_	103.9900	1	103.9900	10758.0100	<0.0001	Significant
X_2_	18.5600	1	18.5600	1920.3200	<0.0001	Significant
X_3_	5.3200	1	5.3200	550.7400	<0.0001	Significant
X_1_X_2_	0.1271	1	0.1271	13.1400	0.0151	Significant
X_1_X_3_	7.6300	1	7.6300	789.4300	<0.0001	Significant
X_2_X_3_	4.2200	1	4.2200	436.5300	<0.0001	Significant
X_1_^2^	44.8800	1	44.8800	4642.8000	<0.0001	Significant
X_2_^2^	3.1500	1	3.1500	325.8600	<0.0001	Significant
X_3_^2^	6.4200	1	6.4200	664.6200	<0.0001	Significant
**Residual**	0.0483	5	0.0097			
Lack of Fit	0.0434	3	0.0145	5.8200	0.1502	Not significant
Pure Error	0.0050	2	0.0025			
**Cor Total**	190.1400	14				
**Std. Dev.**	0.0983	**R**^**2**^**=**		0.9997		
**Mean**	95.8000	**Adj. R**^**2**^**=**		0.9993		
**C.V. %**	0.1026	**Pred. R**^**2**^**=**		0.9963		
		**Adeq. Precision**		138.8737		

An R^2^ value of a regression model over 0.9 indicates a highly significant correlation [49]. In this study, the R^2^ value (coefficient of determination) of the BBD model is 0.9997, suggesting that 99.97 % of dye removal percentage variations could be described by the model, while only 0.03 % cannot be explained. The high values of both R^2^ and adjusted R^2^ (0.9997 and 0.9993, respectively) indicating the model is high significance with excellent fitness for the experimental data.

The predicted R^2^ value of 0.9963 and adjusted R^2^ value of 0.9993, with a difference of less than 0.2, indicating a strong agreement between the two [50], confirming the model's validity in predicting dye removal. The Adequate Precision value of this model is 138.874, indicating an adequate signal and this model can be used to navigate the design space. Adequate precision assesses the signal-to-noise ratio, which must be higher than 4 [51]. Additionally, the lower C.V. (coefficient of variation percentage) value (0.1026 %), implies the accuracy and reliability of the conducted experiments [52,53].

The following second-order polynomial quadratic equation was calculated based on analysis of variance (ANOVA) of the experimental data in order to describe MB dye removal percentage in terms of the coded factors:

(17) Y = + 98.85 + 3.61 × _1_ + 1.51 × _2_ − 0.8158 × _3_ − 0.1782 × 1 × _2_ + 1.38 × 1 × _3_ + 1.03 × 2 × _3_ − 3.49 × _1_^2^ − 0.9236 × _2_^2^ − 1.32 × _3_^2^

Where Y is the predicted dye removal percentage (%), X_1_, X_2_ and X_3_ are the coded levels of the agitation speed (rpm), pH and biomass dose (mg/L), respectively.

### 3.4 Three-dimensional response surface and contour plots

**Fig. 3:**
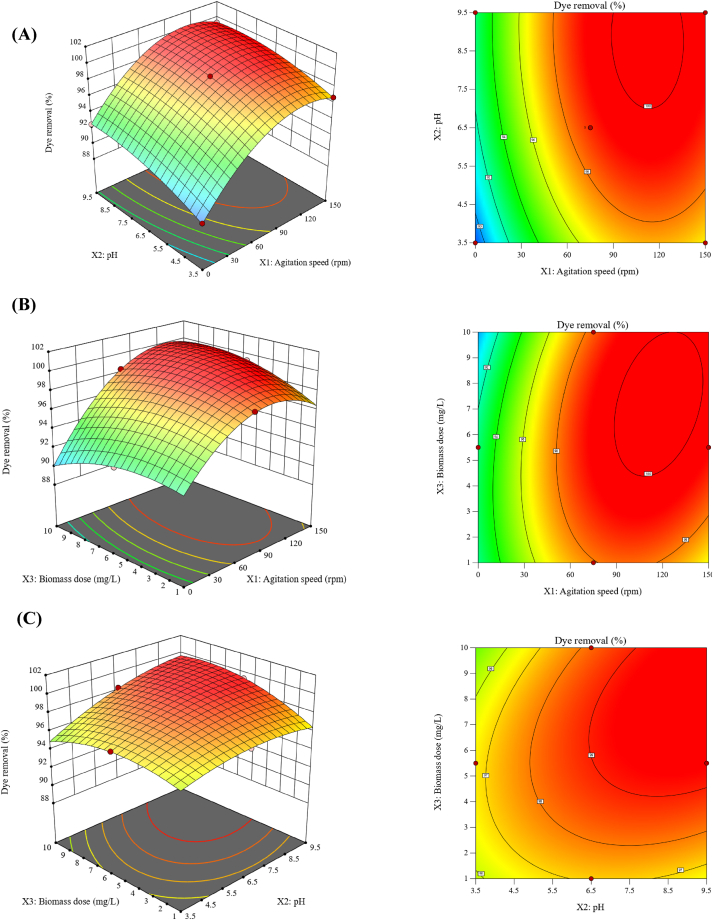
3D response surface and contour plots showing the interactions between each two factors on dye removal by *Gracilaria bursa-pastoris*.

Three-dimensional (3D) surface plots and their corresponding contour plots in Fig. 3 were generated by plotting the dye removal percentage (response results) versus the combination of each two factors (X_1_X_2_, X_1_X_3_ and X_2_ X_3_) while holding the third factor constant at its mid-value (level 0), in order to investigate the effect of individual factors and their interaction on the prediction of dye removal, and to determine the optimum conditions for MB removal.

Fig. 3A illustrates the combined effect of agitation speed (X_1_) and pH (X_2_) on dye removal percentage, while the biomass dosage (X_3_) of the dried *Gracilaria bursa-pastoris* was set at zero level (5.5 mg/L). It's clear that, the dye removal percentage (Y) significantly increases with the increase in the agitation speed. The maximum predicted value of the response (dye removal percentage) of 99.68 % was obtained at agitation speed of 132 rpm. After this value, the dye removal percentage does not increase with the increasing in agitation speed. Similarly, the increase in pH values leads to a gradual increase in dye removal. The maximum dye removal percentage (Y) was obtained at agitation speed of 132 rpm and pH 7.

The interaction effect of both agitation speed (X_1_) and biomass dose (X_3_) of *Gracilaria bursa-pastoris* on MB dye removal percentage was presented in Fig. 3B. The initial pH of the solution (X_3_) was set at zero level (6.5). Agitation speed has a highly significant positive effect on dye removal, while the biomass dose shows a lesser effect on the percentage of dye removal. The maximum dye removal percentage (Y) was achieved with an agitation speed of 132 rpm and a biomass dose of 7.5 g/L.

The dye removal increases significantly with the agitation speed, because of the rate of dye molecule diffusion from the bulk liquid to the liquid boundary layer around the algal biomass particles increases as the agitation speed increases, driven by enhanced turbulence and a thinner liquid boundary layer [54]. Nevertheless, the increase in dye removal with the increase in agitation speeds could be only up to a particular limit, after that, there is no notable increase in dye removal [55,56].

Results also showed that, the further increase in biomass dose doesn't lead to increase in dye removal. This might be due to the saturation of active sites on the biomass surface, which means that additional biomass does not contribute to additional adsorption of dye molecules. Additionally, as the biomass dose increases, the size of the adsorbent particles also increases due to more clumping, resulting in fewer active sites available for adsorption on the adsorbent surface [57,58]. Similar trends were obtained in previous reports [57,59,60].

Fig. 3C shows the simultaneous effect of initial pH (X_2_) and *Gracilaria bursa-pastoris* biomass dose (X_3_) on MB dye removal percentage, while the agitation speed (X_3_) of *Gracilaria bursa-pastoris* was maintained at zero level of 75 rpm. Dye removal slightly increases with the increase in pH. Similarly, the increase in biomass dose leads to a slight increase in the response. The maximum dye removal percentage (Y) was obtained at pH 7 and a biomass dose of 7.5 g/L.

The maximum removal of MB at pH 7 was also achieved by Tahir et al. using *Sargassum* species and *Ulva lactuca* [61]. El-Naggar and Rabei reported that the maximum MB removal by *Gracilaria* seaweed biomass was achieved at pH 8 [27], while at Hammud et al. found that the *Carolina* sp. achieved the highest MB dye removal at a pH of 6.8 [62].

The comparison of MB dye removal efficiency obtained in this study using the dried biomass of *Gracilaria bursa-pastoris* with that of other algal species reported in the literature, is presented in Table 7.

**Table 7:** Comparison of the various algal biomass for MB removal efficiency.

Adsorbent	Dye concentration (mg/L)	Adsorbent dose (g/L)	pH	Contact time (min)	Temperature (°C)	MB removal (%)	References
*Gracilaria corticata*	150	5	8	240	30	94.90	[63]
*Sargassum duplicatum*	20	1	5	70	25	88.90	[64]
*Sargassum latifolium*	40	0.1	10	120	50	95.97	[65]
*Bracteacoccus* sp.	15	60	7	60	30	96.00	[66]
*Gracilaria*	20	6	8	180	30	94.86	[27]
*Chlamydomonas variabilis*	56.40	1.50	7	30	25	80.80	[67]
*Gracilaria bursa-pastoris*	100	7.50	7	240	35	99.68	This study

### 3.5 Model's validity confirmation

The desirability function (DF) was utilized determine the optimal predicted parameters for achieving the highest maximum dye removal (response) [68].

**Fig. 4:**
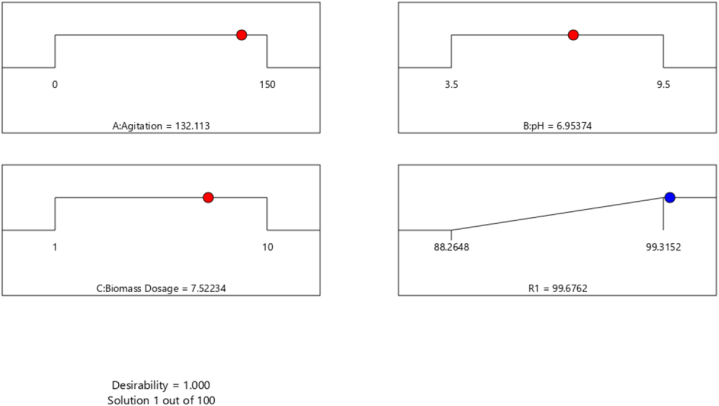
Desirability plot shows the optimum predicted values for the maximum MB dye removal by *Gracilaria bursa-pastoris* (dye concentration: 100 mg/L, temperature: 35 °C, contact time: 4 h, agitation speed: 150 rpm).

Desirability plot in Fig. 4 shows the optimum predicted conditions for maximum MB dye removal. The maximum predicted dye removal of 99.68 % was obtained at the condition of 132.1 rpm, 6.95 and 7.5 g/L for the agitation speed, pH and biomass dose, respectively. Under these optimum conditions, the model was validated by conducting a laboratory confirmation experiment to compare between the experimental and the predicted value of maximum dye removal percentage. The obtained result of 99.4 % came within the confidence interval, confirming a high degree of the model accuracy for the optimization process.

**Fig. 5:**
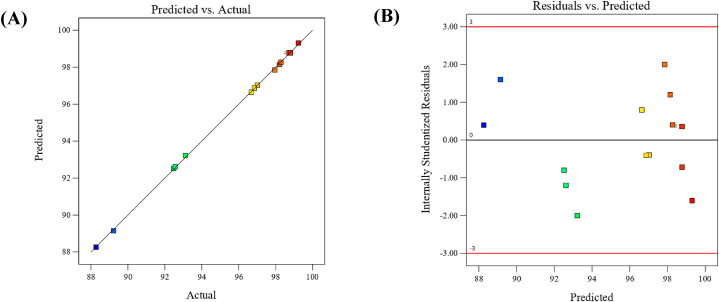
(**A**) Predicted values versus actual values plot; (**B**) Residual versus predicted response values plot. For MB dye removal by *Gracilaria bursa-pastoris*.

In Fig. 5A the experimental (actual) values were plotted versus predicted values. The figure indicated that, the actual and predicted values are linearly correlated and all points are located directly on the 45-degree straight line, which also confirms the accuracy of the model [13].

Additionally, Fig. 5B displays the residuals plotted against the predicted response values. It is evident that the residuals are evenly distributed, with a similar number of points lying both above and below the horizontal line. It indicates a random distribution of residuals values, with an equal number of points falling above and below the horizontal line. Also, the residual values fall in the range of ±3.00 which serves as the standard boundary for recognizing outliers [69].

### 3.6 Characterization of the biosorbent

**Fig. 6:**
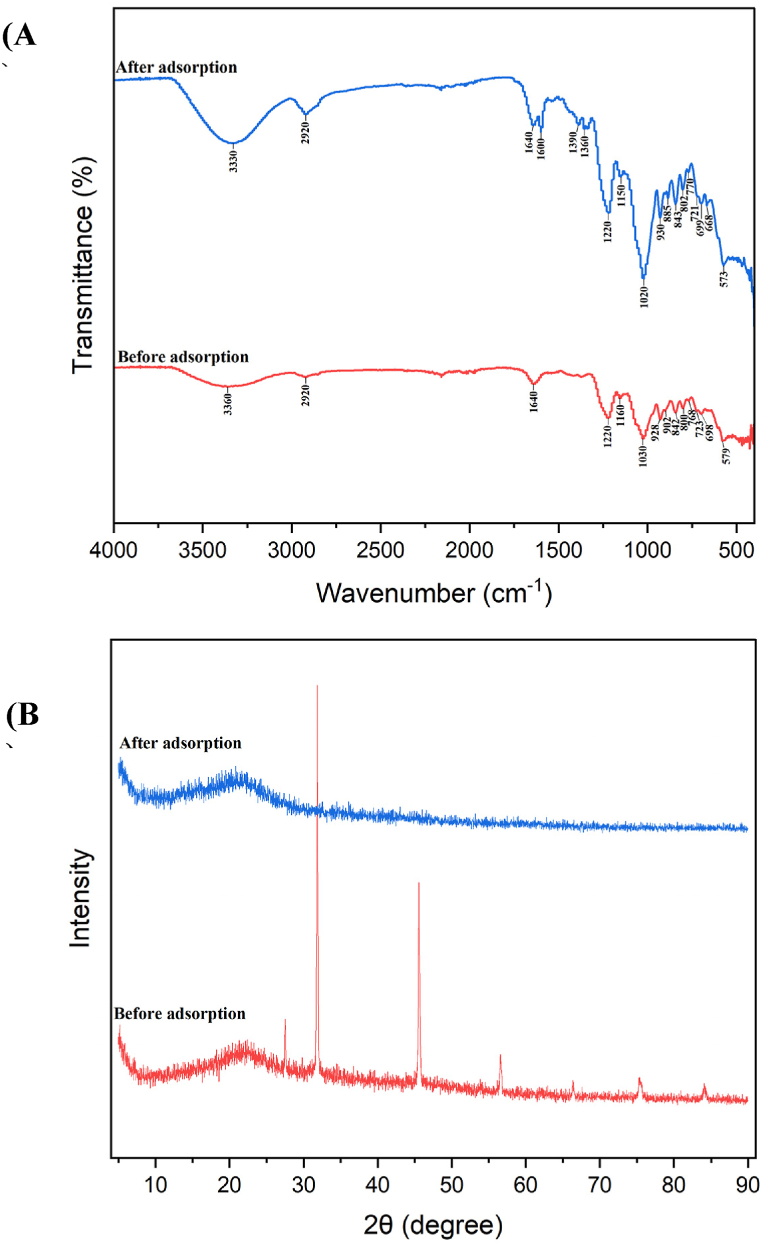
FTIR spectra (**A**); and XRD spectra (**B**) of *Gracilaria bursa-pastoris* dried biomass before and after dye adsorption.

The dried *Gracilaria bursa-pastoris* biomass was analyzed before and after the adsorption of MB dye using FTIR spectroscopy within the wave number range of 400–4000 cm^−1^ (Fig. 6A), to study the alterations in the surface functional groups caused by the interaction of dye ions with those functional groups. The degree of shift in the band indicates how much the functional groups are interacting with the dye ions that have been adsorbed [70].

Fig. 6A illustrates that the FTIR spectra exhibited multiple adsorption peaks, suggesting the presence of diverse functional groups on the surface of the dried *Gracilaria* biomass [13]. The FTIR wavelength of 3360 cm^−1^ corresponds to the O–H stretch and N–H stretch of alcohol and aliphatic primary amine functional group, respectively. This adsorption peak was shifted to the wavelength of 3330 cm^−1^ after adsorption of MB dye, confirming the involvement of the hydroxyl functional group and hydrogen bond in the dye adsorption [[71], [72], [73]]. The given wavelength peaks at 2920 cm^−1^ and 1640 cm^−1^ are associated with the C–H stretching and C=C stretching group, respectively [74,75]. The peaks at wavenumbers of 1600 cm^−1^ (corresponding to C=C stretching of alkene), 1390 cm^−1^ (C–H bending of aldehyde), and 1360 cm^−1^ (associated with S=O stretching of the sulfonate group) appeared in the spectrum after MB dye adsorption. The peak at 1220 cm^−1^ which related to C–O stretching [76] was showed in the FTIR spectra for before and after dye adsorption The adsorption peak at 1160 cm^−1^ (C–O stretching of tertiary alcohol) was changed to 1150 cm^−1^ after dye adsorption. Similarly, the spectrum peak of 1030 cm^−1^ (attributed to C–N stretching of amine group) was shifted to 1020 cm^−1^ after dye adsorption. The intense peaks at the wavelengths of 928, 902, 842, 800, 768, 723 and 698 cm^−1^ for the IR spectrum before adsorption were shifted after adsorption to 930, 885, 842, 803, 770, 721 and 699 cm^−1^. The adsorption peaks in the 700–900 cm^−1^ region of the IR spectrum are distinctive for C–H bond in aromatic compounds [74,77]. The new peaks at 668 cm^−1^ associated with the C–Cl bond functional group [78] appeared in the IR spectrum of *Gracilaria bursa-pastoris* after MB dye adsorption.

Overall, based on the FTIR analysis, the presence of new adsorption peaks, alterations in adsorption intensity, and shifts in peak wave number of the functional groups after dye adsorption, confirming the dye ion interaction with active binding sites on the surface of the adsorbent (*Gracilaria bursa-pastoris*).

Fig. 6B illustrates the XRD spectrum of the dried *Gracilaria bursa-pastoris* biomass before and after dye adsorption are shown in Fig. 6B.

XRD pattern of *Gracilaria bursa-pastoris* before dye adsorption exhibits presence of sharp peaks, which indicate its crystalline nature. These sharp peaks are correspond to various crystalline organic molecules in the marine algae [79].

The main wide peak in the range of around 10° and 30° 2 θ degrees in the XRD pattern, before and after dye adsorption, is related to amorphous structures of cellulose and polysaccharides [13,80].

XRD results showed both amorphous and crystalline structure of the row biomass of *Gracilaria bursa-pastoris*. Similar structural patterns were reported in *Gracilaria edulis* [25], *Gracilaria* crassa [79] and *Gracilaria corticate* [81].

**Fig. 7:**
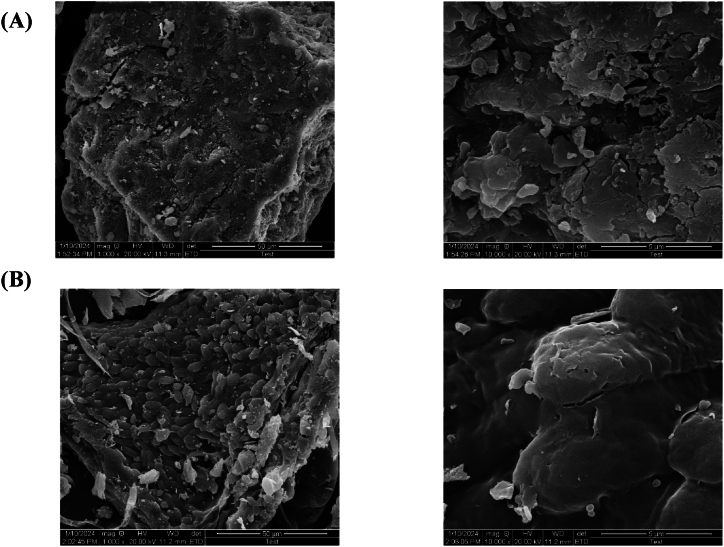
Scanning electron microscopy (SEM) images of *Gracilaria bursa-pastoris* before (**A**); and after (**B**) dye adsorption at magnifications of×1000 and × 10000.

The morphology surface of *Gracilaria bursa-pastoris* before and after dye adsorption was investigated using SEM images at magnifications of 1000 × and 10000 × (Fig. 7).

The SEM images before dye adsorption (in Fig. 7A) illustrate that the biomass surface of *Gracilaria bursa-pastoris* was porous and unoccupied. Whereas after dye adsorption (Fig. 7B), the surface was filled with dye molecules and the porous surface texture of the biomass disappeared, leading the surface to appear irregular [27,28].

### 3.7 Adsorption isotherm investigations

**Fig. 8:**
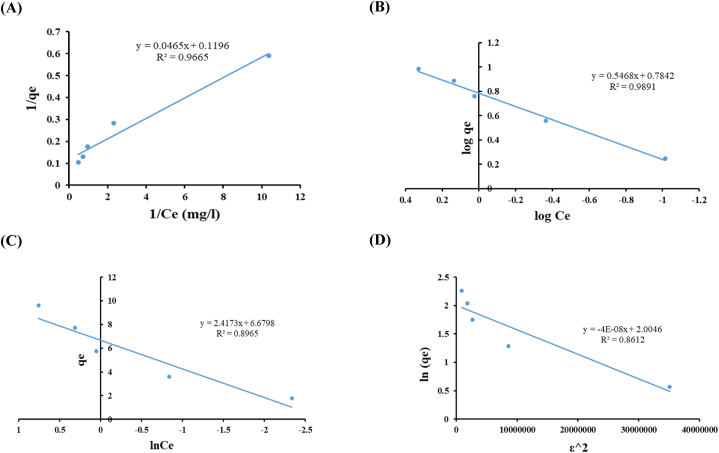
Adsorption isotherm model of Langmuir (**A**); Freundlich (**B**); Temkin (**C**); and Dubinin–Radushkevich model (**D**) (contact time: 4 h, biomass dosage: 10 g/L, pH: 7.5, agitation speed: 150 rpm).

Langmuir, Freundlich, Temkin and Dubinin-Radushkevich isotherm models (Fig. 8 and Table 8) were employed in this study to describe the process of adsorption on the surfaces.

**Table 8:** Adsorption isotherm models constants**.**

Langmuir model					Freundlich model			
Q_m_ (mg/g)	K_L_	R_L_	R^2^	SSE	K_F_	1/n	R^2^	SSE
8.3612	2.5720	0.0090	0.9665	0.0053	6.0842	0.5468	0.9891	0.0037
**Temkin model**				**Dubinin-Radushkevich model**				
**B (J/mol)**	**A (L/g)**	**R**^**2**^	**SSE**	**q**_**s**_**(mg/g)**	**K**_**DR**_**(mol**^**2**^**/kJ**^**2**^**)**		**R**^**2**^	**SSE**
2.4173	15.8521	0.8965	4.0515	7.4233	4.3 × 10^−8^		0.8612	0.2520

According to the Langmuir model theory, the key assumption is that adsorption process takes place at distinct and uniform locations on the adsorbent, therefore, it assumes that once a dye ion has been adsorbed at a specific site, no further sorption can occur at that particular site [36].

The obtained results in Table 8 show that, the predicted value of Q_m_ (maximum adsorption capacity) calculated from the Langmuir isotherm was 8.36 mg/g. In previous works, the maximum adsorption capacity obtained by the red seaweed *Pterocladia capillacea* was 5.714 mg/g for synthetic dye solution [28], 3.306 mg/g by the brown seaweed *Sargassum wightii* [82], 5.23 mg MB/g by *Caulerpa racemosa* var. *cylindracea* [83], and 40.2 mg MB/g by *Ulva lactuca* [84].

The dimensionless separation factor (R_L_) was used to further describe the interaction of dye ions onto the adsorbent (R_L_). The R_L_ value, as defined by Hall et al. (1966) [85], indicates the favorability (0 < R_L_ < 1), un favorability (R_L_ > 1), linearity (R_L_ = 1), or irreversibility (R_L_ = 0) of the isotherm model. The R_L_ value of 0.009 (in the range of 0–1), indicating that the adsorption of MB by the dried biomass of *Gracilaria bursa-pastoris* was favorable.

Freundlich isotherm model suggests that the adsorption process take place on heterogeneous surfaces with different sites having different adsorption energies, and it is also employed to study the adsorption equilibrium in non-monolayer surfaces [37].

By plotting Log q_e_ against Log C_e_ (as shown in Fig. 8B), the Freundlich model constants; K_F_ and n, can be calculated from the intercept and slope, respectively. The K_F_ values give an indication about the amount of adsorbed dye, while 1/n value represents the adsorption intensity or heterogeneity of the adsorbent surface. In Table 8, the calculated values of K_F_ and 1/n were found to be 6.0842 mg/L and 0.5468, respectively. The value of n should be in the range of 1–10 [86]. In this study it was found to be 1.829 (1/n = 0.5468) indicating a favorable adsorption process.

The Temkin isotherm model assumes that the interaction between the sorbent and the sorbate leads to a decrease in the heat of adsorption. According to the obtained data, the Temkin isotherm constant (B) was found to be 2.41 J/mol, which indicates the release of heat during the adsorption reaction and the adsorption process is exothermic since B value > 0 [87].

The adsorption data was also fitted to Dubinin–Radushkevich isotherm (Fig. 8D). This model is used to differentiate between physical and chemical adsorption. The calculated E value (free energy of adsorption) was found to be 3.4 kJ/mol demonstrating the physical sorption mechanism. E value lower than 8 kJ/mol indicates the Physisorption mechanism [88].

According to the presented data in Table 8, the best-fitted model is the Freundlich model with the highest coefficient (R^2^ = 0.9891) and the lowest sum square error (SSE = 0.0037) compared to the Langmuir model with R^2^ = 0.9665 and SSE = 0.0053. Whereas the Temkin model recorded values of 0.8965 and 4.0515 for R^2^ and SSE, respectively, and Dubinin-Radushkevich model showed values of 0.8612 and 0.252 for R^2^ and SSE, respectively.

It can be clearly said that, the Freundlich model is the best-fitted isotherm model for explaining the adsorption of MB dye onto *Gracilaria bursa-pastoris* biosorbent, indicating that the adsorption process occurs onto heterogeneous surface of the adsorbent, and there might exist multiple kinds of sorption sites on the surface [89].

### 3.8 Adsorption kinetic investigations

**Fig. 9:**
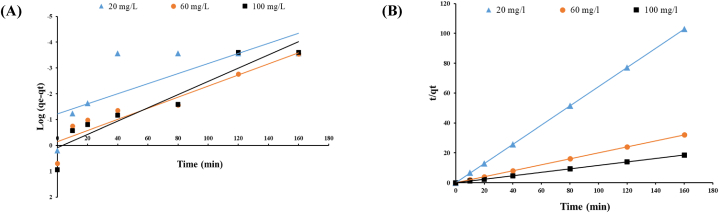
(**A**) Pseudo-first-order model; (**B**) Pseudo-second -order model for MB adsorption on *Gracilaria bursa-pastoris*. (dye concentration: 20, 60 and 100 mg/L, biomass dosage: 10 g/L, pH: 7.5, agitation speed: 150 rpm).

Lagergren's pseudo-first-order model and Ho's pseudo-second-order model were used in this study to describe the adsorption mechanism of MB dye onto the dried biomass of *Gracilaria*. These two kinetic models and their related parameters are presented in Fig. 9 and Table 9 for 3 dye concentrations (20, 60 and 100 mg/L).

**Table 9:** Kinetic parameters of pseudo-first-order model and pseudo-second -order model for MB adsorption onto *Gracilaria bursa-pastoris* under different dye concentrations.

Dye concentration (mg/L)	Pseudo-first-order				Pseudo- second -order				Experimental qe (exp.) (mg/g)
	k_1_ (min^−1^)	qe (calc.) (mg/g)	R^2^	SSE	K_2_ (g/mg· min)	qe (calc.) (mg/g)	R^2^	SSE	
**20**	0.0449	0.0595	0.5954	5.6939	3.4697	1.5569	0.9999	0.0499	1.5547
**60**	0.0495	0.7154	0.8912	1.2457	0.6126	5.0000	0.9999	0.0083	4.9917
**100**	0.0590	1.2179	0.8911	1.7717	0.4257	8.6356	0.9999	0.0016	8.6218

From Table 9, the R^2^ values of pseudo-first-order model are 0.5954, 0.8912 and 0.8911 for the dye concentrations of 20, 60 and 100 mg/L, respectively. On the other hand, R^2^ values of pseudo-second -order model is 0.9999 for all dye concentrations. Additionally, pseudo-second -order model showed lower SSE values of 0.0499, 0.0083 and 0.0016 for the concentrations of 20, 60 and 100 mg/L, respectively. Moreover, all values of q_e_ (cal.) (calculated equilibrium adsorption capacity) obtained from Ho's model were almost equal to those of experimented (qe (exp.)) in all dye concentrations. From these results, it's obvious that adsorption process of MB dye onto the dried biomass of *Gracilaria bursa-pastoris* follows the pseudo second-order kinetic model and seems to be controlled by chemical mechanisms [28]. Numerous studies indicated that the pseudo-second-order model was more suitable for describing the kinetics of sorbate-solute interactions [28,63,90,91].

### 3.9 Adsorption thermodynamics

**Fig. 10:**
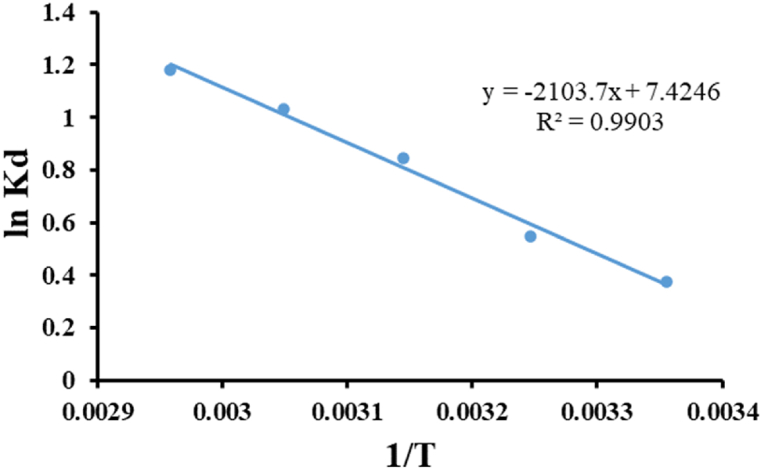
Van't Hoff plot for MB adsorption on *Gracilaria bursa-pastoris*. (dye concentration: 100 mg/L, biomass dosage: 10 g/L, contact time 4 h).

Thermodynamics was applied to determine whether the adsorption process is spontaneous or non-spontaneous and exothermic or endothermic to understand the interactions between the adsorbent and sorbate and the nature of the adsorption process. Fig. 10 shows the Van't Hoff plot of ln K_d_ versus 1/T which used to calculate the values of ΔH° (enthalpy changes) and ΔS° (entropy changes) from the slope and intercept, respectively. The calculated thermodynamics parameters ΔH°, ΔS°, and ΔG° are presented in Table 10. The negative value of ΔG° (−0.933 to −3.310 K J/mol) signifies the spontaneity and feasibility of the adsorption process, and the decrease of ΔG° values with increasing temperature indicates that the adsorption is more favorable at higher temperatures. The positive value of ΔH° (17.490 K J/mol) indicates an endothermic nature of the adsorption process [92]. This means that energy is absorbed from the surroundings during the adsorption process, strongly suggesting the adsorption of dye molecules onto the adsorbent surface is favored at higher temperatures. The positive value of ΔS° (61.728 J/mol K) indicates an increase in randomness at the solid/liquid interface during the adsorption process, reflecting the affinity of the adsorbent towards the adsorbate [93].

**Table 10:** Thermodynamic parameters for MB adsorption onto *Gracilaria bursa-pastoris*.

Temp. (K)	Kd	ΔG° (K J/mol)	ΔH° (KJ/mol)	ΔS° (J/mol K)
**298**	1.482	−0.933	17.490	61.728
**308**	1.762	−1.407		
**318**	2.373	−2.231		
**328**	2.852	−2.814		
**338**	3.302	−3.310		

## 4 Conclusions

In the current study, four different seaweeds were investigated for their ability to remove MB dye from aqueous solutions. Among the four examined algae, *Gracilaria bursa-pastoris* recorded the highest dye removal percentage. PBD and BBD for the response surface methodology were applied as statistical approaches to study the effect of different environmental conditions on MB removal from aqueous solutions using *Gracilaria bursa-pastoris* dried biomass, in order to achieve the maximum dye removal percentage. Using PBD, agitation speed, pH and biomass dose were found to be the significant parameters affecting dye removal. These three parameters were further optimized using BBD. The other three factors; dye concentration (100 mg/L), temperature (35 °C) and time (4 h) which were insignificant on dye removal, were maintained at their high levels of PBD (which exerted a positive effect on dye removal). The maximum predicted dye removal of 99.68 % was achieved at agitation speed of 132.1 rpm, pH 6.95 and biomass dose of 7.5 g/L, and the experimental dye removal percentage was 99.4 %. The dried *Gracilaria bursa-pastoris* biomass was analyzed before and after the adsorption of MB dye using FTIR, SEM and XRD, demonstrating the participation of several functional groups in the adsorption process and changes in the cell surface morphology following the dye adsorption. Freundlich isotherm model (R^2^ = 0.9891) was a better fit than Langmuir (R^2^ = 0.9665), Temkin (R^2^ = 0.8965), and Dubinin-Radushkevich (R^2^ = 0.8612) models for describing MB dye adsorption onto *Gracilaria bursa-pastoris* biosorbent. The adsorption kinetics were best described by the pseudo-second-order model (R^2^ = 0.9999), suggesting the chemical interactions between dye ions and the algal biomass. The thermodynamic parameters of ΔG° (−0.933 to −3.310 K J/mol), ΔH° (17.490 K J/mol) and ΔS° (61.728 J/mol K) indicated that the adsorption process was spontaneous, feasible and endothermic. In the future, further studies are needed to address other environmental pollutants and industrial wastewater treatment, as well as to improve the process from a small lab-scale to a larger pilot scale.

## Data availability statement

Data are contained within the article.

## CRediT authorship contribution statement

**Ali Borham:** Writing – review & editing, Writing – original draft, Validation, Methodology, Investigation, Formal analysis, Conceptualization. **Mohammed Haroun:** Visualization, Investigation. **Ibrahim A. Saleh:** Visualization, Funding acquisition. **Naser Zomot:** Software, Funding acquisition. **Mohammad K. Okla:** Writing – review & editing, Validation. **Mofeed Askar:** Writing – review & editing, Software. **Mohamad Elmasry:** Writing – review & editing, Software. **Abdelmonem Elshahat:** Resources, Investigation. **Lei Liu:** Writing – original draft, Formal analysis. **Chen Zhao:** Resources, Formal analysis. **Juanjuan Wang:** Writing – original draft, Validation, Methodology. **Xiaoqing Qian:** Validation, Supervision, Project administration, Funding acquisition, Conceptualization.

## Declaration of competing interest

The authors declare that they have no known competing financial interests or personal relationships that could have appeared to influence the work reported in this paper.
